# Erratum: Docetaxel-Loaded Mixed Micelles and Polymersomes Composed of Poly (caprolactone)-Poly (ethylene glycol) (PEG-PCL) and Poly (lactic acid)-Poly (ethylene glycol) (PEG-PLA): Preparation and In-vitro Characterization [Iran J Pharm Res. 2019;18(1): e126114]

**DOI:** 10.5812/ijpr-154205

**Published:** 2024-08-27

**Authors:** Editor-in-Chief IJPR

**Affiliations:** 1 School of Pharmacy, Shahid Beheshti University of Medical Sciences, Tehran, Iran

It has come to our attention that Table 4 in the above-mentioned article were published incorrectly due to an error from the previous publisher (SBMU) and an oversight by the authors, who did not thoroughly review their article when receiving the Galley Proof. 

Please note, the new publisher (Brieflands) started with this journal from January 1, 2022 (https://brieflands.com/posts/new_publisher_ijpr), while the old publisher (SBMU) was responsible for publications until December 2021. This article was originally published in January 2019.

We deeply regret this error and any confusion it may have caused. The correct version of Table 4 is now provided below.

Correct Table 4: 

**Table 4.** Polymerosomes Formulation Contains DTX with Different Proportions of CL15 and LA15

No.	CL15%	LA15%	Z-Average (nm)	PdI	Zeta potential (mV)	DTX (µg/ml)	EE%	LC%
**P1 ** ^**a**^	100	0	120.7 ± 7.4	0.151 ± 0.04	-10.4	143.6 ± 0.25	71.8 ± 0.04	0.71 ± 0.02
**P2 ** ^**b**^	0	100	151.3 ± 3.4	0.164 ± 0.02	-13.5	133.5 ± 0.03	66.6 ± 0.32	0.66 ± 0.01
**P3 ** ^**c**^	50	50	131.6 ± 6.3	0.39 ± 0.034	-16.2	143 ± 0.71	71.5 ± 0.45	0.71 ± 0.05
**P4 ** ^**d**^	25	75	97.73 ± 10.2	0.31 ± 0.08	-12.8	135.1 ± 0.25	67.55 ± 0.42	0.67 ± 0.04
**P5 ** ^**e**^	75	25	140.7 ± 8.4	0.3 ± 0.042	-17.0	177 ± 0.38	93.5 ± 0.56	0.93 ± 0.07

^a^ P1: Polymersome contains 100 % CL15 and 0 % LA15.

^b^ P2: Polymersome contains 0 % CL15 and 100 % LA15.

^c^ P3: Polymersome contains 50 % CL15 and 50 % LA15.

^d^ P4: Polymersome contains 25 % CL15 and 75 % LA15.

^e^ P5: Polymersome contains 75 % CL15and 25 % LA15.

We apologize for any inconvenience this may have caused to the readers and the scientific community. We appreciate your understanding and thank you for your continued support.

Sincerely,

The Head of Ethics Committee

Ethics Committee of Brieflands Publishing, On Behalf of Dr. Elham Rezaee (Editor-in-Chief)

